# Supplementary material for: Cloud-Based and Locally Deployed Language Models in Nursing and Health Care: An AI Act–Aligned Framework
Source: JMIR Med Inform. 2026 Sep 25;14:e90854. doi: 10.2196/90854 (PMC13618209; doi:10.2196/90854)
Supplement: Supplementary file 1 [file medinform-v14-e90854-s001.docx]

**Multimedia Appendix 1.** Technical specifications of Large and Small Language Models evaluated in the study.

| **Model Name and Provider** | **Context Window (Tokens)** | **License / Access** | **Architecture Description** | **Training & Alignment Approach** | **Training Data Transparency and Primary Sources** |
| --- | --- | --- | --- | --- | --- |
| **DeepSeek-R1** DeepSeek AI | 128K | MIT License; open weights; commercial use permitted. | Mixture-of-Experts reasoning model based on DeepSeek-V3-Base; 671B total parameters and 37B activated parameters. | Cold-start supervised data followed by a multi-stage pipeline comprising two reinforcement-learning stages and two supervised fine-tuning stages; includes reasoning and human-preference alignment. | Weights, parameter counts, context length, training stages, evaluation settings, and technical report are public. The complete sample-level pretraining corpus is not released.  Primary sources: [DeepSeek-R1 repository](https://github.com/deepseek-ai/DeepSeek-R1); [technical report](https://arxiv.org/abs/2501.12948) |
| **Claude 3.7 Sonnet** standard mode Anthropic | 200K | Proprietary; commercial API/service. | The same proprietary hybrid-reasoning model used in both study conditions. In standard mode it produces near-instant responses. Exact parameter count and low-level architecture are not disclosed. | Anthropic reports pretraining on diverse data, human-feedback post-training, and Constitutional AI. The standard-mode condition does not activate extended thinking. | Broad data-source categories and safety methods are reported, but exact datasets, parameter count, weights, and the model-specific optimization recipe are not public.  Primary sources: [Claude 3.7 release](https://www.anthropic.com/news/claude-3-7-sonnet); [Anthropic Transparency Hub](https://www.anthropic.com/transparency) |
| **Claude 3.7 Sonnet** extended thinking mode Anthropic | 200K | Proprietary; commercial API/service. | Same underlying Claude 3.7 Sonnet model, with extended thinking enabled. It self-reflects before answering; API users could set a thinking budget. Exact parameter count and low-level architecture are not disclosed. | Same documented model-development approach as standard mode: diverse pretraining data, human feedback, and Constitutional AI. Extended thinking is an inference mode, not a separately disclosed base model. | Broad data-source categories and safety methods are reported, but exact datasets, weights, parameters, and the model-specific optimization recipe are not public.  Primary sources: [Claude 3.7 release](https://www.anthropic.com/news/claude-3-7-sonnet); [Anthropic Transparency Hub](https://www.anthropic.com/transparency) |
| **Claude 3 Opus** Anthropic | 200K | Proprietary; commercial API/service. | Proprietary multimodal large language model supporting text and image inputs. Exact parameter count and low-level architecture are not disclosed. | Anthropic publicly documents Constitutional AI and safety tuning for the Claude family; a complete Opus-specific post-training recipe is not disclosed. | Capabilities, context window, model card, and safety evaluations are public; exact training corpus, parameters, weights, and full training recipe are not.  Primary sources: [Claude 3 family release](https://www.anthropic.com/news/claude-3-family); [Constitutional AI](https://www.anthropic.com/research/constitutional-ai-harmlessness-from-ai-feedback) |
| **OpenAI GPT-4o** OpenAI | 128K | Proprietary; commercial API/service. | A single end-to-end multimodal neural model trained across text, vision, and audio. Exact parameter count and low-level architecture are not disclosed. | OpenAI reports training-data filtering, behavioral refinement through post-training, automated and human evaluations, and external red teaming. The exact optimization recipe is not public. | Input/output modalities, context window, safety methods, and evaluations are documented. Exact datasets, data mixture, parameter count, and weights are not public.  Primary sources: [GPT-4o release](https://openai.com/index/hello-gpt-4o/); [OpenAI model page](https://platform.openai.com/docs/models/gpt-4o) |
| **OpenAI o1-preview** OpenAI | 128K | Proprietary; commercial API snapshot (later deprecated). | Reasoning model in the OpenAI o1 family. Exact parameter count and low-level architecture are not disclosed. The model was a real released checkpoint, not a speculative name. | Large-scale reinforcement learning for chain-of-thought reasoning, with deliberative alignment. OpenAI reports public, partnership-derived proprietary, and in-house data categories. | High-level training-data categories, filtering, safety evaluations, and alignment approach are documented. Exact corpus, parameter count, weights, and full recipe are not public.  Primary sources: [OpenAI o1 System Card](https://openai.com/index/openai-o1-system-card/); [o1-preview model page](https://platform.openai.com/docs/models/o1-preview) |
| **Gemini 2.0 Pro Experimental** Google DeepMind | 2M | Proprietary; experimental commercial API/service. | Experimental multimodal Gemini model with text output, structured output, function calling, search, and code-execution tools. Google did not publicly disclose parameter count or whether this checkpoint was dense or MoE. | No checkpoint-specific pretraining or alignment recipe was publicly disclosed for Gemini 2.0 Pro Experimental. Therefore, no RLHF, MoE, or dataset claim should be inferred from another Gemini version. | Capabilities and 2-million-token context were published. Exact architecture, parameter count, weights, training corpus, and post-training recipe were not disclosed.  Primary sources: [Google release and official comparison chart](https://developers.googleblog.com/en/gemini-2-family-expands/) |
| **Microsoft Copilot** consumer service used via browser | Not publicly fixed; depends on the routed model and service configuration. | Proprietary consumer service. | Not a single fixed language model. Microsoft documents an orchestration pipeline in which classifiers process the prompt, an SLM determines whether web grounding is needed and which language model should answer, and Bing results may ground the response. | System messages align responses with Microsoft AI Principles; input/output classifiers and web grounding are applied. The training and alignment of the routed foundation model vary and are not disclosed for each request. | Service-level processing is documented, but the exact foundation-model checkpoint, context window, training data, and routing decision used in the March 2025 tests cannot be reconstructed from the manuscript.  Primary sources: [Microsoft Copilot Transparency Note](https://support.microsoft.com/en-us/microsoft-copilot/transparency-note-for-microsoft-copilot) |
| **Grok-2** xAI | Not publicly disclosed in xAI's Grok-2 release documentation. | Proprietary; commercial service. | Proprietary text-and-vision model. xAI did not disclose the exact parameter count or low-level architecture for Grok-2. | xAI reports an AI-Tutor preference process: evaluators compare two model responses for instruction following and factual accuracy. The complete pretraining and post-training recipe is not disclosed. | Release status, capabilities, and preference-evaluation process are public. Context length, full training corpus, parameters, weights, and optimization details are not.  Primary sources: [xAI Grok-2 beta release](https://x.ai/news/grok-2) |
| **Qwen2.5-Max** API ID: qwen-max-2025-01-25 Alibaba Cloud | 32,768 (max input 30,720; max output 8,192) | Proprietary; commercial API/service. | Large-scale Mixture-of-Experts model. Alibaba/Qwen did not disclose total or activated parameter counts for this commercial checkpoint. | Pretrained on more than 20 trillion tokens, then post-trained with curated supervised fine-tuning and reinforcement learning from human feedback. | Exact snapshot ID, context limits, broad token scale, MoE design, SFT, and RLHF are documented. Weights, parameter counts, full corpus, and detailed training recipe are not public.  Primary sources: [Qwen2.5-Max announcement](https://qwenlm.github.io/blog/qwen2.5-max/); [Alibaba Model Studio model list](https://www.alibabacloud.com/help/en/model-studio/models) |
| **Llama 3.3 70B Instruct** Meta | 128K | Open weights under the custom Llama 3.3 Community License. | 70B-parameter text-only autoregressive transformer with Grouped-Query Attention. | Pretrained on more than 15 trillion tokens from a new mix of publicly available online data. The instruction-tuned model uses supervised fine-tuning and reinforcement learning with human feedback. | Weights, architecture class, parameter count, context, token scale, knowledge cutoff, broad data category, SFT, and RLHF are documented; exact item-level corpus is not.  Primary sources: [Meta model card](https://huggingface.co/meta-llama/Llama-3.3-70B-Instruct) |
| **Mistral Large 2** version 24.07 / mistral-large-2407 Mistral AI | 128K | Open weights under Mistral Research License for research/noncommercial use; commercial self-deployment requires a commercial license. | Dense 123B-parameter language model with native function calling and JSON output. | Mistral reports a large proportion of code and multilingual data, improved instruction following, function-calling training, and training to acknowledge uncertainty. A specific RLHF claim is not publicly substantiated for this checkpoint. | Weights, parameter count, context, languages, license, and selected training emphases are public; the full corpus and complete post-training algorithm are not.  Primary sources: [Mistral release](https://mistral.ai/news/mistral-large-2407/); [official model card](https://huggingface.co/mistralai/Mistral-Large-Instruct-2407) |
| **Perplexity Sonar** Perplexity AI | 128K in current official Sonar documentation; exact March 2025 endpoint/snapshot was not recorded. | Proprietary; commercial API/search service. | Web-grounded, non-reasoning search-answer model/service optimized for real-time retrieval and citations. Current API documentation does not disclose the exact underlying checkpoint or parameter count. | Retrieval grounding and citation generation are documented. The exact foundation-model post-training and alignment method for the tested March 2025 service snapshot are not publicly disclosed. | Service behavior and current context limit are documented, but weights, corpus, architecture, parameter count, and exact historical snapshot are not. The manuscript should report the API/model identifier if logs exist.  Primary sources: [current Sonar model documentation](https://docs.perplexity.ai/docs/sonar/models/sonar); [Perplexity changelog](https://docs.perplexity.ai/docs/resources/changelog) |
| **Gemma 2** Google exact checkpoint size not reported in manuscript | 8,192 for the Gemma 2 family | Open weights under the Gemma Terms of Use. | Decoder-only transformer family released in 2B, 9B, and 27B variants, using alternating local sliding-window and global attention, GQA, RoPE, RMSNorm, GeGLU, and logit soft-capping. The tested size cannot be identified from the manuscript. | 2B and 9B use knowledge distillation; 27B was trained from scratch. Instruction-tuned checkpoints use supervised fine-tuning followed by RLHF. The applicable details depend on the unidentified tested checkpoint. | Weights and technical report are public, including token scales and broad sources (web documents, code, science articles). Exact sample-level corpus is not public, and the manuscript omits the checkpoint size/version.  Primary sources: [Gemma 2 technical report](https://storage.googleapis.com/deepmind-media/gemma/gemma-2-report.pdf); [Gemma Terms](https://ai.google.dev/gemma/terms) |
| **OpenAI o1-mini** OpenAI | 128K | Proprietary; commercial API model. | Faster o1-family reasoning model, particularly effective for coding. Exact parameter count and low-level architecture are not disclosed. | Large-scale reinforcement learning for chain-of-thought reasoning, with deliberative alignment. OpenAI reports public, partnership-derived proprietary, and in-house data categories. | High-level data categories, filtering, safety evaluations, and alignment approach are documented. Exact corpus, parameter count, weights, and full recipe are not public.  Primary sources: [OpenAI o1 System Card](https://openai.com/index/openai-o1-system-card/); [o1-mini model page](https://platform.openai.com/docs/models/o1-mini) |
| **Qwen2.5-14B-Instruct** Alibaba Cloud / Qwen | 32,768 in the default configuration; up to 131,072 with the documented YaRN extension; max generation 8,192. | Apache License 2.0; open weights. | Causal transformer: 14.7B total parameters (13.1B non-embedding), 48 layers, GQA with 40 query and 8 key/value heads, RoPE, SwiGLU, RMSNorm, and QKV bias. | Model card reports pretraining and post-training/instruction tuning. It does not provide enough checkpoint-specific evidence to label the exact post-training algorithm as RLHF. | Weights, configuration, architecture, parameter counts, license, and deployment guidance are public. The complete raw training corpus and detailed post-training dataset/recipe are not.  Primary sources: [official Qwen model card](https://huggingface.co/Qwen/Qwen2.5-14B-Instruct) |

**Notes.** (a) Context window denotes the provider/model maximum documented for the named release; it may differ from the effective limit imposed by ChatHub. (b) Claude 3.7 Sonnet standard and extended-thinking conditions are listed separately because the study evaluated them separately, although they use the same underlying model. (c) The exact tested checkpoint cannot be reconstructed for Microsoft Copilot, Perplexity Sonar, or Gemma 2 unless original API/browser logs identify the service mode or model ID. (d) Current Perplexity documentation reports a 128K Sonar context, but the manuscript did not preserve the March 2025 endpoint identifier; the historical configuration is therefore not asserted as identical. (e) “Open weights” is used where weights are available under model-specific terms; it is not treated as synonymous with an OSI-approved open-source license.
