# Supplementary material for: Cloud-Based and Locally Deployed Language Models in Nursing and Health Care: An AI Act–Aligned Framework
Source: JMIR Med Inform. 2026 Sep 25;14:e90854. doi: 10.2196/90854 (PMC13618209; doi:10.2196/90854)
Supplement: Supplementary file 2 [file medinform-v14-e90854-s002.docx]

**Multimedia Appendix 2. Glossary of technical terms, prompt engineering formulas tailored to the nursing field, and cross-cultural adaptability of the framework.**

**Multimedia Appendix 2 comprises the following sections:**

- **A.1.** A glossary of fundamental terms in the field of Generative AI
- **A.2.** A summary of prompt engineering formulas tailored to the nursing field
- **A.3.** Cross-cultural and global adaptability of the framework

**A.1. Glossary of fundamental technical terms adopted in the manuscript**

**Jailbreaking** refers to the technique of crafting specialized prompts designed to elude the safety and ethical filters built into a Language Model, attempting to elicit a response that the model is programmed to refuse (e.g., providing harmful instructions). The jailbreak techniques adopted in this study are: 1) Framing in vector subspace by means of fictitious scenarios (distancing), 2) Creating an apparently legitimate purpose, 3) Context engineering - Providing situational pressures that may justify unethical actions, 4) Misdirection - Focusing on character development or narrative rather than unethical advice, and 5) Complexity - Adding nuances that blur the ethical boundaries. *LLM jailbreak prompts* are transparently included **in the Zenodo repository (10.5281/zenodo.17727785).**

**Fine-tuning** (i.e., specialization) in healthcare involves the adaptation of a pre-trained, generalist Large Language Model (LLM) for a specific clinical domain through additional training on a curated, specialized dataset. This methodology transforms the model from a generalist system into a specialized decision-support tool tailored to a particular area of practice. For instance, an LLM intended for adoption in Oncology Nursing could be fine-tuned utilizing a corpus of Evidence-Based Nursing (EBN) protocols for managing chemotherapy-related toxicities, patient education materials, and clinical practice guidelines from professional bodies such as the Oncology Nursing Society (ONS) or the Infusion Nurses Society (INS). The expertise of specialist nurses is therefore indispensable for curating this training literature and these datasets to ensure the model's alignment with state-of-the-art clinical standards. Accordingly, research proposals for such projects should include, within their business plan, a request for funding to secure copyrights for any proprietary source materials used.

**Transformers** represent the core architecture of LLMs. LLMs are models designed to understand, generate, and interpret language efficiently. Based on the principles of machine learning (ML), LLMs are trained on large datasets consisting of textual sources, enabling them to identify patterns, nuances, and structures inherent in natural language. The Transformer architecture was introduced by Vaswani et al. (2017) in their article entitled “Attention Is All You Need”[1]. Unlike traditional models that process data sequentially (recurrent neural networks), one element at a time, transformers use an attention mechanism called “*Multi-Head Attention*”, in which each head is an individual neural network that assigns weights to each element of each sentence in relation to all other elements, allowing for the simultaneous consideration of all words in the sentence. This capability gives the transformer the property of *“scalability*”, i.e. the ability to analyse large data sets not only more quickly, but also more effectively: it improves the model's ability to understand the context and relationships within the text, resulting in more coherent responses. Therefore, the larger the context, expressed as the number of tokens that can be processed by an LLM, the greater its ability to understand, in parallel, the correlations between sections of the nursing record that are located at a long radius of distance from each other.

The **“*inference phase*”** can be defined as the “interactions” between the user and the model, regardless of whether they occur directly with the provider, or with API intermediation. The inference phase stands in contrast to the *“training phase”* of a model.

**MoEs and eco-sustainability:** the green advantage emerges during large-scale daily use (during the inference phase), where continuous computational efficiency compensates for the initial energy investment. MoEs require greater total parameters and more resources during initial training. However, instead of activating the entire parameter network for each token processed, as occurs with traditional dense LLMs, their gating network routes the inputs only to a small subset of experts. As a practical utility consideration in the nursing field, a prompt that clearly establishes a “persona” (e.g., “You are a nurse specialising in wound care...”) provides robust semantic context initially. This facilitates the gating network in making more effective routing decisions. During inference, they selectively activate only a fraction of the total parameters for each input, drastically reducing energy consumption (2-4 times less) and consequently diminishing the requirement for water utilized for cooling data centres.

**Long Context-LLMs (LC-LLM)** are characterized by the capacity to analyze a huge number of Context Window (Tokens). These technical specifications are shown in Multimedia Appendix 2. One token corresponds to approximately 0.75 words. Before the release of LC – LLMs, in order to attach extensive documentation to a prompt, it was necessary to use RAG (retrieval augmented generation), i.e., the compression of text into vector representation. Therefore, the release of LC models and their technological advancement [2] represented a breakthrough.

- **A.2. A summary of prompt engineering formulas tailored to the nursing field**

Enhanced clarity and precision in prompt formulation correlate directly with increased accuracy of model responses. In structuring prompts for healthcare-focused LLMs, our methodology commenced with establishing a specific "persona" such as “*Act as the expert* IBD care manager in a gastroenterology clinic including the conducting of clinical trials”. or “*You are a researcher excelling in* generative AI applied to Nursing” to augment response accuracy, by limiting the model’s focus to specific clinical/research/managerial subdomains, while reducing informational noise. This systematic approach to prompt engineering incorporates multiple fundamental principles essential for optimal clinical AI implementation.

*When using Mixture of Experts (MoEs), the specification of the persona is also more fundamental: this facilitates the gating network in making more effective routing decisions, activating only the pertinent “expert”network for each processed token.*

We provided detailed clinical cases, requesting specific outputs such as “*Provide detailed nursing techniques* for the management of [specific condition]”. In cases of incomplete prompts, “*Ask questions before answering…”* prompts the model to seek missing data, thereby reducing potential bias. This methodology encourages the model to inquire about omitted details, such as age, comorbidities, polypharmacotherapy, previous treatments, and any omitted vital parameter, etc. Subsequently, priority is placed on responses grounded in high-evidence, peer-reviewed sources, *explicitly requiring rationales and biometric parameter cutoffs for each procedural step* of the personalized clinical algorithm, following multiparametric analysis. Asking an LLM to think step by step means guiding it towards a Chain of Thought (CoT), which not only increases their problem-solving capacity but elicits advanced reasoning [3], thereby making the rationale underlying each specific procedure or clinical indication more comprehensible.

The formula for obtaining the answer and the associated bibliography in appropriate format was refined to the following optimal structure for evaluation purposes:

“Base your response exclusively on high-evidence sources. Ensure adherence to updated nursing best practices documented in peer-reviewed journals and official guidelines with the highest level of evidence classification, prioritizing patient safety. Incorporate in-text citations in standard APA format (Author, Year) throughout the text body. At the end, create a subchapter reporting APA-style references for all cited studies, including DOIs whenever possible and hyperlinks for online sources. Provide only authentic references; you can use Crossref API to resolve unmatched references online (online search required). Do not provide fabricated references.

FORMAT. Use bold for key concepts. Write the body of the answer with a word count close to 600 words, without bullet points, while maintaining readability. However, format the reference list ensuring a clear distinction between each citation and link.”

Finally, **delimiters** serve a dual function: they allow both to make the model understand where the instruction ends and where the data begins, and to reduce the risk of prompt injection attacks, i.e. an imput containing instructions to execute malicious commands [4]. For the first function, square brackets are sufficient, as in the examples provided in the dataset. For instance: analyze the clinical case in [clinical case]. Conversely, the most reliable delimiters with respect to the criticality of prompt injections are XML Tags or triple quotes. For instance: analyze the clinical case in <*clinical case****>***. The rationale resides in the fact that such delimiters constitute, for the model, instructions to treat the content within them exclusively as data and not as executable instructions. “By clearly demarcating user-provided content from the core prompt instructions, delimiters ensure that the user input is treated strictly as data to be processed rather than executable logic” [4].

In automated healthcare contexts, such as customer services in the health sector, such attacks could lead to harmful responses, putting patient safety at risk.

Among the parameters to be set, a very low temperature (the range across different providers can vary from 0.0 to 2***,*** where setting 0.0 causes the model to select the token with the highest probability at each step, namely greedy decoding), variably increases the likelihood of a deterministic response, but this is not equivalent to ensuring an improvement in the accuracy of the personalized clinical care procedural algorithms, which should balance different features (for instance, vital parameters or specific conditions) nor does it ensure the model categorically refuses responses on grounds of uncertainty.

Li et al. (2025) proposed a BERT-based temperature selector to identify the optimal temperature for a given prompt for different models grouped by size from 1 to 80 Billions of parameters, demonstrating, as a novelty, that the cutoff at which statistical significant improvements in performance occur increases with model size [5]. However, this does not cancel out at all the possibility of hallucinations [6].

The adoption of prompt engineering strategies is vital for accuracy augmentation and risk mitigation, particularly considering the LLMs’ “closed box” nature, although rare exceptions are emerging, such as Apertus LLM [7].

However, it is does not constitute true algorithmic explainability of the internal reasoning pathways that each model follows to provide its clinical decision-support outputs, thus representing a central challenge of Explainable AI (XAI).

**A.3.Cross-cultural and global adaptability of the framework**

The proposed methodological workflow, from model selection to evaluation is relevant especially for non-Western and LMIC nursing contexts. In the contemporary international context, marked by geopolitical tensions, including hybrid threats such as cyberwarfare, a robust framework, aligned with EU regulation 2024/1689 was essential. The 27 proposed items include in fact, the structured evaluation for all safety and security dimensions, emphasizing the necessity for redirecting future research on local functioning models contemplating also operational resilience, eco-sustainability, and robust privacy protection for sensitive data processed through LMs during the interactions, and operational resilience, also in in the absence of internet.

Attention was also placed in the assessment of anti-discrimination, adaptability to local policies, as well as in the *innovative structured approach for clinical adversarial testing, that is adaptable to any field of nursing.*

For example, regarding the typical real-world case in the context of vaccinations at a newly opened vaccination centre, **clinical adversarial testing**, which asks teachers to point out BIAS (specifically **ethno-physiological bias)** to students, could consist of asking them to specify the appropriate needle length for an IM deltoid injection for a 37-year-old male patient of South Asian origin: *a top-performance model will* *proactively identify the missing information* and will respond by asking for the patient's BMI before giving an answer, or at least will classify the needles based on each hypothetical BMI. Conversely, inadequate training, based on datasets where socio-demographic data may be biased towards *Western data (Fenech-Borg et al., 2025), could emphasize data on local reactogenicity and immunogenicity focused mainly on the risk of muscle hypopenetration - i.e. < 5 mm - (due to a BMI higher than Western standards) rather than needle hyperpenetration* [8]. Regarding the importance of adjusting each feature for multiparametric interventions, including needle length selection, to the typical BMI characterizing a specific ethnicity, the author N. Shankar (2014) underlines, indeed, how in a sample of 200 Indian subjects with a body mass index (BMI) mean of 24.2, the universal use of a 1 inch (25 mm long) needle caused overpenetration in 50% of the vaccinated subjects, hitting the bone or periosteum, despite the precaution of leaving outside 3 mm of the needle closest to the hub [9].

This approach moves beyond simple guideline adherence checking, teaching nurses the high-level critical thinking and expert oversight required to challenge AI outputs, thereby ensuring the safe and equitable integration of LLM-based platforms into both academic and clinical practice. Tests probing for bias are essential in each nursing specialization considering that most **AI models** are not only trained on English datasets but ***may be trained with non-homogeneous socio-demographic, clinical, ethno-physiological and psychosocial variables [10].*** These types of testing move beyond simple guideline checking and guide academic monitoring to the high-level critical thinking required to challenge AI outputs, ensuring the safe and equitable integration of AI globally.

**Integrated references**

Chen, B., Zhang, Z., Langrené, N., & Zhu, S. (2025). Unleashing the potential of prompt engineering for large language models. *Patterns*, *6*(6).

Fenech-Borg, E. Z., Meznaric-Kos, T. P., Lekovic-Bojovic, M. D., & Hentze-Djurhuus, A. J. (2025). The Cultural Gene of Large Language Models: A Study on the Impact of Cross-Corpus Training on Model Values and Biases. *arXiv preprint arXiv:2508.12411*.

Li, L., Sleem, L., Nichil, G., & State, R. (2025). Exploring the impact of temperature on large language models: Hot or cold? *Procedia computer science*, *264*, 242-251.

Omar, M., Sorin, V., Collins, J. D., Reich, D., Freeman, R., Gavin, N., Charney, A., Stump, L., Bragazzi, N. L., & Nadkarni, G. N. (2025). Multi-model assurance analysis showing large language models are highly vulnerable to adversarial hallucination attacks during clinical decision support. *Communications medicine*, *5*(1), 330.

Swiss, A. I. I. (2025). swiss-ai/Apertus-70B-Instruct-2509 [Text generation model (LLM)]. *Hugging Face*. <https://huggingface.co/swiss-ai/Apertus-70B-Instruct-2509>

Wei, J., Wang, X., Schuurmans, D., Bosma, M., Xia, F., Chi, E., Le, Q. V., & Zhou, D. (2022). Chain-of-thought prompting elicits reasoning in large language models. *Advances in neural information processing systems*, *35*, 24824-24837.

1.	Vaswani A, Shazeer N, Parmar N, Uszkoreit J, Jones L, Gomez AN, et al. Attention is all you need. Advances in Neural Information Processing Systems. 2017;30.

2.	Li Z, Li C, Zhang M, Mei Q, Bendersky M. Retrieval augmented generation or long-context LLMs? A comprehensive study and hybrid approach. Proceedings of the 2024 Conference on Empirical Methods in Natural Language Processing: Industry Track. 2024:881-893. doi:10.18653/v1/2024.emnlp-industry.66.

3.	Wei J, Wang X, Schuurmans D, Bosma M, Xia F, Chi E, et al. Chain-of-thought prompting elicits reasoning in large language models. Advances in Neural Information Processing Systems. 2022;35:24824-24837. doi:10.52202/068431-1800.

4.	Chen B, Zhang Z, Langrené N, Zhu S. Unleashing the potential of prompt engineering for large language models. Patterns. 2025;6(6):101260. doi:10.1016/j.patter.2025.101260 PMID:40575123.

5.	Li L, Sleem L, Nichil G, State R. Exploring the impact of temperature on large language models: hot or cold? Procedia Computer Science. 2025;264:242-251. doi:10.1016/j.procs.2025.07.135.

6.	Omar M, Sorin V, Collins JD, Reich D, Freeman R, Gavin N, et al. Multi-model assurance analysis showing large language models are highly vulnerable to adversarial hallucination attacks during clinical decision support. Communications Medicine. 2025;5(1):330. doi:10.1038/s43856-025-01021-3 PMID:40753316.

7.	Swiss AI Initiative. swiss-ai/Apertus-70B-Instruct-2509. Hugging Face. 2025.

8.	Sblendorio E. Intramuscular vaccine administrations including the adoption of “Zeta-track technique” & “without aspiration slow injection technique” (ZTT & WASiT): a prospective review. Frontiers of Nursing. 2023;10(1):21-42. doi:10.2478/fon-2023-0003.

9.	Shankar N, Saxena D, Lokkur PP, Kumar NM, William NC, Vijaykumar N. Influence of skin-to-muscle and muscle-to-bone thickness on depth of needle penetration in adults at the deltoid intramuscular injection site. Medical Journal Armed Forces India. 2014;70(4):338-343. doi:10.1016/j.mjafi.2014.08.005 PMID:25382907.

10.	Fenech-Borg EZ, Meznaric-Kos TP, Lekovic-Bojovic MD, Hentze-Djurhuus AJ. The Cultural Gene of Large Language Models: A Study on the Impact of Cross-Corpus Training on Model Values and Biases. arXiv. Preprint posted August 17, 2025. arXiv:2508.12411. doi:10.48550/arXiv.2508.12411.
